# Supplementary material for: Climate change may outpace current wheat breeding yield improvements in North America
Source: Nat Commun. 2022 Sep 30;13:5591. doi: 10.1038/s41467-022-33265-1 (PMC9525655; doi:10.1038/s41467-022-33265-1)
Supplement: Supplementary file 1 — Supplementary information [file 41467_2022_33265_MOESM1_ESM.pdf]

Supplementary Information

Supplementary Figures

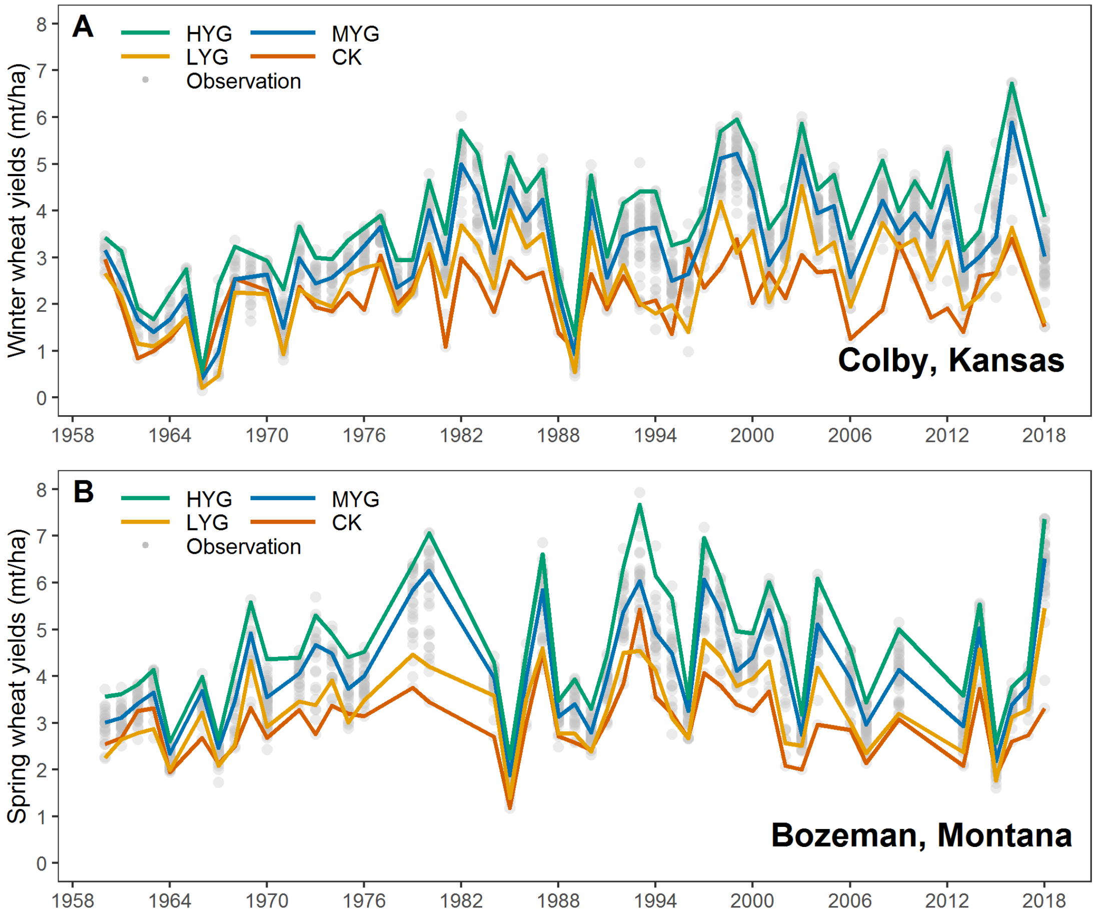

Supplementary Figure 1. A demonstration of yield time series of wheat yields. A, Winter wheat yields in Colby Kansas; **B**, Spring wheat yields in Bozeman Montana. The line's color denotes genotypes: high-yielding genotype (HYG); median-yielding genotype (MYG); low-yielding genotype (LYG); check variety (CK). The background data points show the yield observations of each experimental entry in each year.

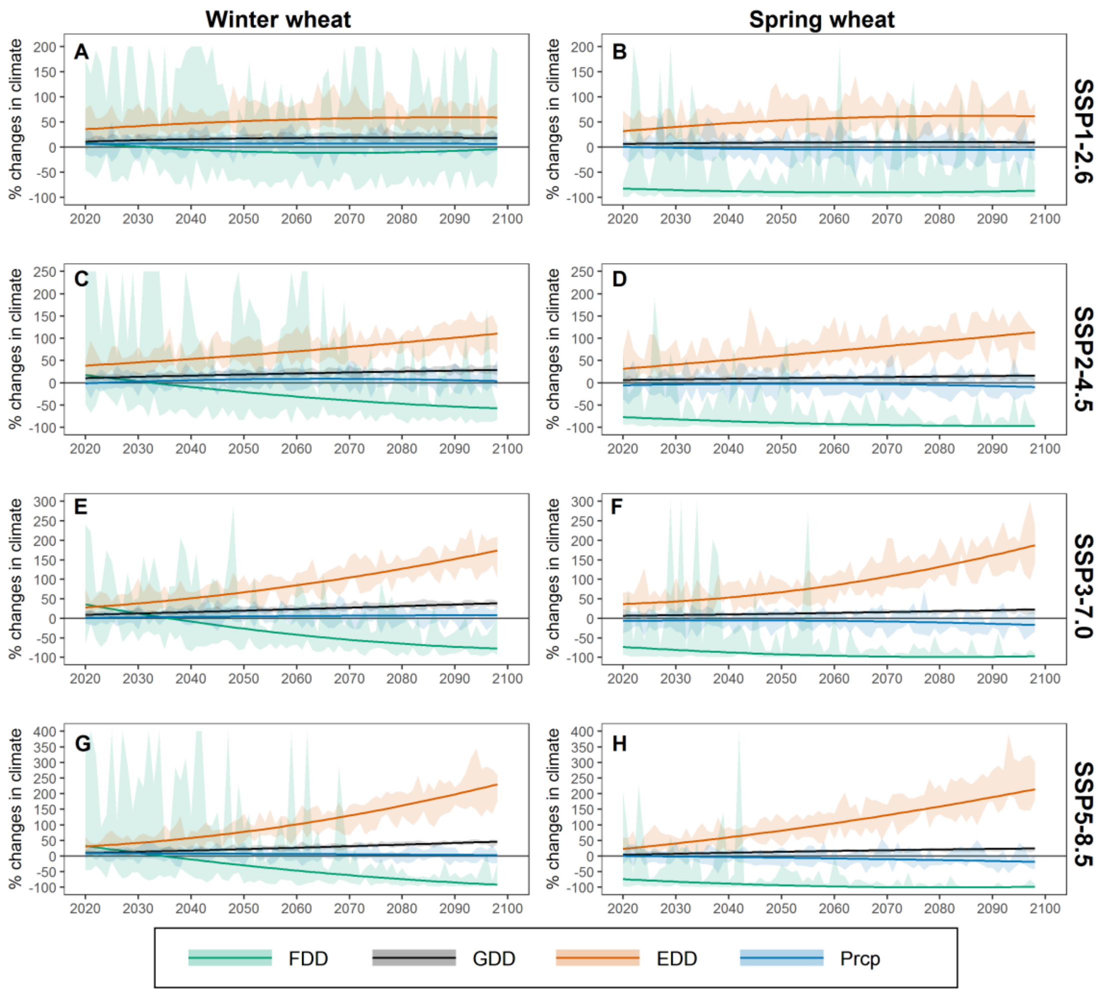

Supplementary Figure 2. The trend of climate variables simulated by climate models under four SSPs. The four climate variables are freezing degree-day (FDD), growing-degree-day (GDD), extreme-growing-degree (EDD) and precipitation (Prcp). The left panel shows the climate trends for winter wheat (A, C, E, G) and the right panel (B, D, F, H) shows spring wheat. The first row shows result for the SSP1-2.6 scenario (A, B), the second for SSP2-4.5 (C, D), the third for SSP3-7.0 (E, F) and the fourth for SSP5-8.5 (G, H). The line are the best fit of median changes in each year, and the shading areas show an estimate of the 95% confidence interval from six climate models.

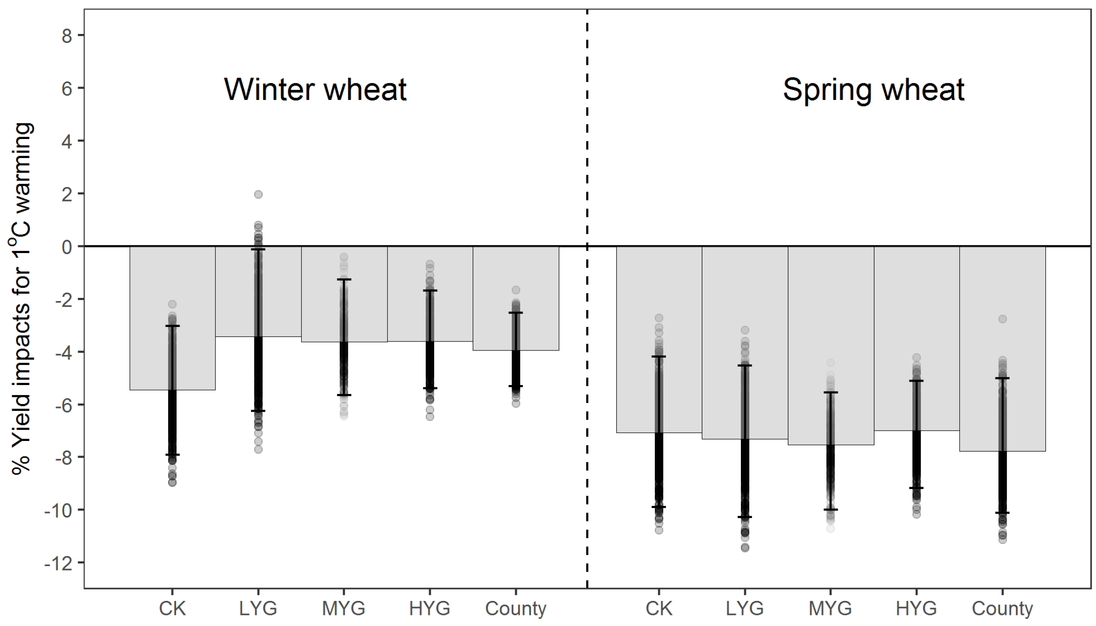

Supplementary Figure 3. Comparison of model estimates of yield impacts of 1°C warming using regression models for data of nurseries and county-level yield statistics from National Agricultural Statistics Service[1] and Statistics Canada[2]. The centers of error bars denote median yield impact, and the error bars show the 95% confidence interval produced by bootstrapping method (n=1,000). The background data points are the yield impacts for each estimation by bootstrap analysis.

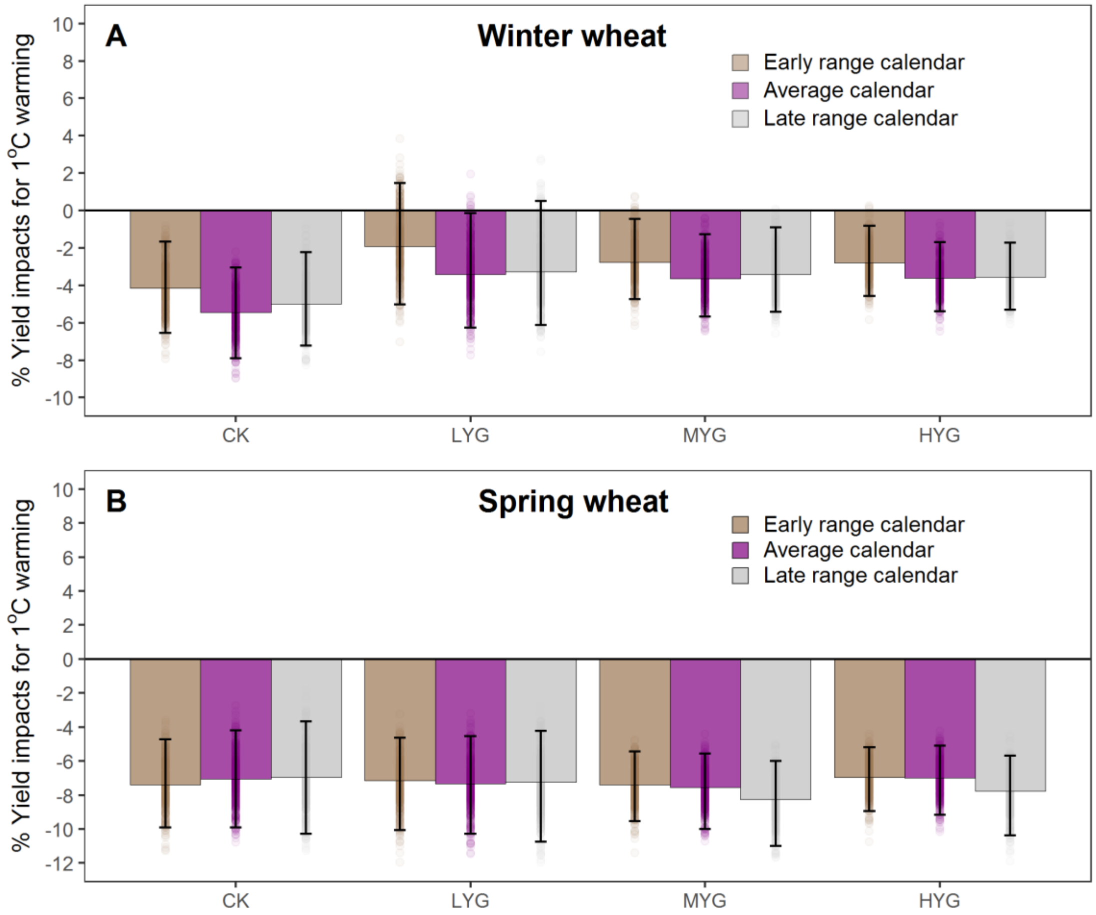

Supplementary Figure 4. Model estimated yield impact for 1°C warming based on various definitions on growing season. **A**, results for winter wheat; **B**, results for spring wheat. The bar's color denotes various definitions on growing season. We calculated climate variables based on Sacks et al.[3]. The results reported in the main paper is derived from the average planting and harvesting dates in each site (purple bars), and we repeated the analysis based on the lower (yellow bars) and upper (grey bars) range of planting and harvesting time windows. The centers of error bars denote median yield impact, and the error bars show the 95% confidence interval produced by bootstrapping method (n=1,000). The background data points are the yield impacts for each estimation by bootstrap analysis.

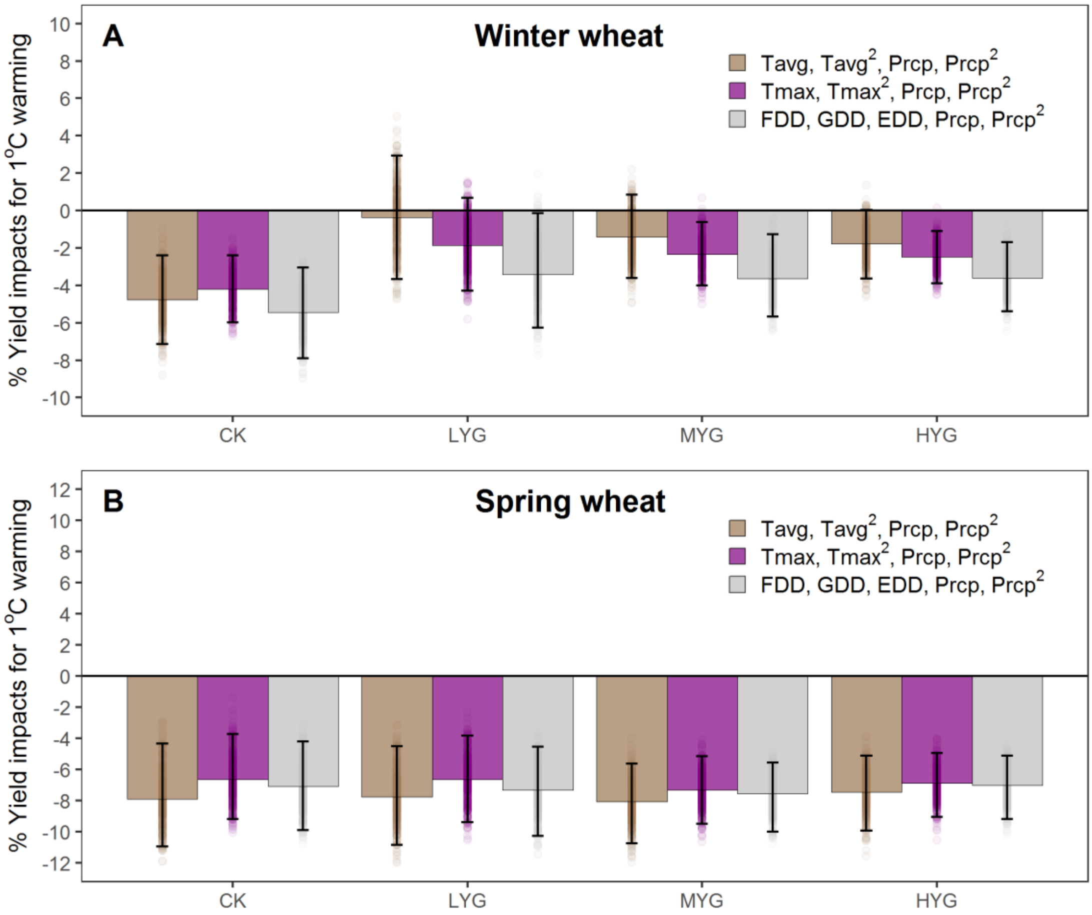

Supplementary Figure 5. Model estimated yield impact for 1°C warming based on various model specifications. **A**, results for winter wheat; **B**, results for spring wheat. The bar's color denotes various model specifications. The centers of error bars denote median yield impact, and the error bars show the 95% confidence interval produced by bootstrapping method (n=1,000). The background data points are the yield impacts for each estimation by bootstrap analysis. The main results in the paper relate to a model with climate variables: freezing degree-day (FDD), growing-degree-day (GDD), extreme-growing-degree (EDD) and precipitation (Prcp). Results were qualitatively similar when replacing the GDD variables with daily maximum temperature (Tmax) and quadratic term for daily maximum temperature ($Tmax^2$) over growing season. However, predicted impacts of warming had greater uncertainties, especially for winter wheat, when the model used daily average temperature (Tavg) and quadratic term for daily average temperature ($Tavg^2$). We also conducted the model with daily minimum temperature (Tmin) and quadratic term for daily minimum temperature ($Tmin^2$), which returns statistically insignificant results (p>0.05). The regression coefficients in these alternative model specifications can be seen in Supplementary Table 3.

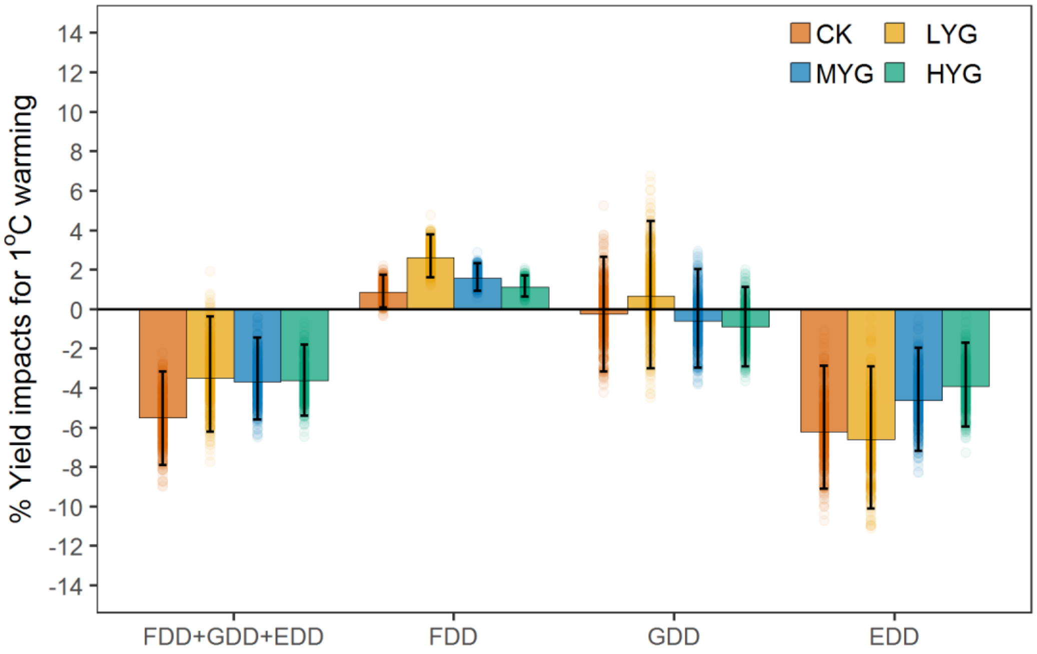

Supplementary Figure 6. Model estimated yield impact of 1°C warming on the four categories of genotypes of winter wheat but using the temperature thresholds for spring wheat from heading to maturity growing phase (Supplementary Table 4). The bar's color denotes genotypes: high-yielding genotype (HYG); median-yielding genotype (MYG); low-yielding genotype (LYG); check variety (CK). The centers of error bars denote median yield impact, and the error bars show the 95% confidence interval produced by bootstrapping method (n=1,000). The background data points are the yield impacts for each estimation by bootstrap analysis.

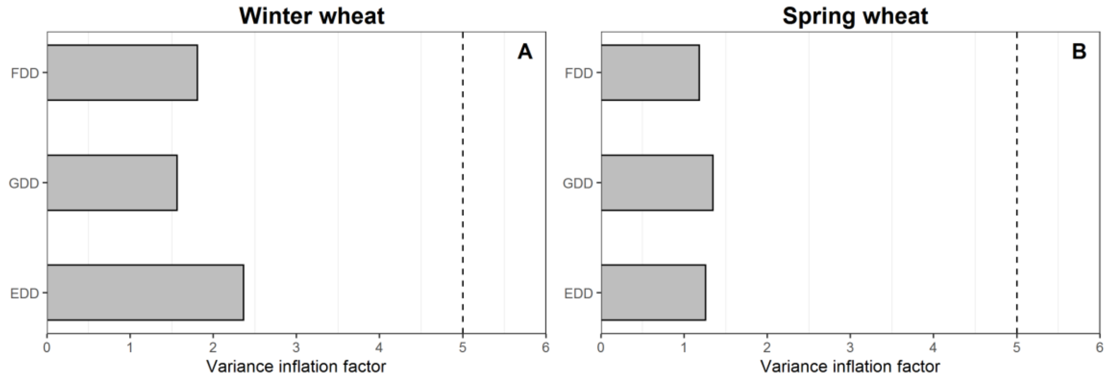

Supplementary Figure 7. Variance inflation factors of FDD, GDD and EDD. A, the results for winter wheat data; **B**, the results for spring wheat data. Based on James et al.[4], regression does not present a serious multicollinearity problem need to be caution if variance inflation factors are less than 5 (dashed line).

Supplementary Tables

Supplementary Table 1. Regression coefficients of winter and spring wheat. The statistical significance of each climate variable is based on the two-tailed *t*-test. The number in parenthesis is the *p*-value

**Variables**	Winter wheat				Spring wheat			
	CK	MYG	HYG	LYG	CK	MYG	HYG	LYG
FDD	-3.80e-04	-6.4e-04	-4.6e-04	-1.05e-03	-2.42e-02	-1.80e-02	-1.86e-02	-1.86e-02
	(0.0266)	(1.15e-05)	(1.23e-04)	(8.92e-07)	(0.0021)	(0.0035)	(0.00108)	(0.0181)
GDD	-5.22e-05	-7.85e-05	-1.0e-05	3.20e-05	4.80e-05	1.46e-04	1.81e-04	1.69e-05
	(0.7137)	(0.5216)	(0.3022)	(0.8585)	(0.8545)	(0.478)	(0.3386)	(0.9486)
EDD	-6.3e-04	-4.7e-04	-4.0e-04	-6.7e-04	-1.57e-03	-1.74e-03	-1.67e-03	-1.56e-03
	(0.000239)	(0.00139)	(0.00102)	(0.00173)	(1.12e-06)	(8.03e-12)	(1.11e-12)	(1.39e-06)
Prcp	2.26e-03	2.79e-03	2.48e-03	2.91e-03	2.42e-03	2.87e-03	2.78e-03	3.03e-03
	(1.68e-11)	(1.10e-21)	(3.23e-25)	(6.64e-12)	(8.98e-05)	(3.35e-09)	(5.67e-10)	(9.89e-07)
$Prcp^2$	-2.52e-06	-2.61e-06	-2.21e-06	-2.88e-06	-5.50e-06	-5.37e-06	-4.86e-06	-6.58e-06
	(6.11e-14)	(2.32e-19)	(1.30e-20)	(9.36e-12)	(3.87e-08)	(9.27e-12)	(2.09e-11)	(5.90e-11)

Supplementary Table 2. Estimated yield response to 1°C warming in previous studies

Regional scale	% Yield response to 1°C warming	Estimation method/model	References
Kansas, USA	(-10.0, -1.0)	Panel-data model	Tack et al.[5]
USA	(-7.0, -3.0)	Panel-data model	Lobell et al.[6]
Mexico	(-12.0, -6.9)	CERES-Wheat model	Lobell et al.[7]
Netherlands, Argentina, India, Australia	(-10.0, -5.0)	Multiple crop models	Asseng, et al.[8]
Global	-3.28%	Panel-data model	Wilcox and Makowski [9]
Global	-6%	Multiple crop models	Asseng, et al.,[10]

Supplementary Table 3. Regression coefficients of winter and spring wheat using alternative model specifications. The statistical significance of each climate variable is based on the two-tailed *t*-test. The number in parenthesis is the *p*-value

**Variables**	Winter wheat				Spring wheat			
	CK	MYG	HYG	LYG	CK	MYG	HYG	LYG
Tavg	0.065	0.046	0.027	0.103	0.0956	0.3123	0.2536	0.378
	(0.0818)	(0.1609)	(0.3111)	(0.0306)	(0.5393)	(0.0109)	(0.0255)	(0.1504)
$Tavg^2$	-0.0071	-0.0037	-0.00278	-0.0066	-0.00477	-0.0108	-0.009	-0.0125
	(0.00676)	(0.1047)	(0.1353)	(0.0468)	(0.2645)	(0.001339)	(0.0038)	(0.00342)
Prcp	0.0024	0.00295	0.002619	0.0031	0.002528	0.0031	0.00303	0.0032
	(3.36e-13)	(2.45e-24)	(4.83e-28)	(6.02e-14)	(5.06e-05)	(2.83e-10)	(3.54e-11)	(1.93e-07)
$Prcp^2$	-2.58e-06	-2.68e-06	-2.27e-06	-2.99e-06	-5.38e-06	-5.42e-06	-4.93e-06	-6.64e-06
	(1.35e-14)	(4.22e-20)	(2.40e-21)	(1.92e-12)	(1.33e-07)	(1.77e-11)	(4.07e-11)	(8.03e-11)
Tmax	0.115	0.1098	0.0822	0.185	0.434	0.630	0.505	0.820
	(0.0226)	(0.0119)	(0.0357)	(0.003838)	(0.0055)	(2.81e-07)	(8.40e-06)	(1.40e-07)
$Tmax^2$	-0.0052	-0.0044	-0.00354	-0.00675	-0.00983	-0.0138	-0.0113	-0.01744
	(0.00313)	(0.00395)	(0.00455)	(0.002559)	(0.00157)	(1.33e-08)	(5.24e-07)	(1.71e-08)
Prcp	0.0022	0.00281	0.002478	0.002991	0.002059	0.00242	0.002386	0.002598
	(3.87e-11)	(1.95e-21)	(1.41e-24)	(3.33e-12)	(0.00133)	(1.39e-06)	(2.88e-07)	(4.26e-05)
$Prcp^2$	-2.46e-06	-2.58e-06	-2.17e-06	-2.85e-06	-4.86e-06	-4.58e-06	-4.17e-06	-5.79e-06
	(2.89e-13)	(1.51e-18)	(1.23e-19)	(2.30e-11)	(2.21e-06)	(1.05e-08)	(1.92e-08)	(1.20e-08)
Tmin	-0.021	0.0122	0.0040	0.0353	-0.1046	-0.053	-0.0544	-0.0726
	(0.1063)	(0.2778)	(0.6621)	(0.0321)	(0.2486)	(0.465)	(0.4158)	(0.422)

$Tmin^2$.	-0.00539	-0.00081	-0.00046	-0.00216	0.00112	-0.00098	-0.00049	-0.00032
	(0.0544)	(0.7381)	(0.81739)	(0.5422)	(0.7766)	(0.7555)	(0.866)	(0.9363)
Prcp	0.00254	0.00298	0.002668	0.003133	0.00304	0.0036	0.0035	0.00364
	(1.42e-14)	(3.69e-25)	(1.86e-29)	(6.43e-14)	(4.88e-07)	(1.13e-13)	(7.38e-15)	(1.91e-09)
$Prcp^2$	-5.32e-06	-2.70e-06	-2.30e-06	-2.97e-06	-5.78e-06	-5.76e-06	-5.28e-06	-6.85e-06
	(5.32e-15)	(2.34e-20)	(8.09e-22)	(2.26e-12)	(9.96e-09)	(9.01e-13)	(1.63e-12)	(1.27e-11)

Supplementary Table 4. The base and optimum growing temperature in each phenological phases for winter and spring wheat. The threshold is based on Narciso et al.[11]; Shroyer et al.[12], Porter and Gawith[13], Tack et al.[5] and Saiyed et al.[14].

Wheat	Month	Growing phases	T_{frez} (°C)	T_{base} (°C)	T_{opt} (°C)
Winter wheat	Sep	Planting	0.0	3.0	7.5
	Oct	Vegetative	0.0	3.0	7.5
	Nov	Hardening	-5.0	3.0	7.5
	Dec	Dormant	-11.0	0.0	5.0
	Jan	Dormant	-11.0	0.0	5.0
	Feb	Dormant	-11.0	0.0	5.0
	Mar	Vegetative	-4.0	1.5	10.6
	Apr	Jointing	-4.0	5.0	18.5
	May	Heading	-1.0	5.0	20.0
	Jun	Filling	-2.2	5.0	20.7
	Jul	Maturity	-2.2	5.0	23.5
Spring Wheat	April	Planting	-8.0	3.0	7.5
	May	Tillering	0.0	3.0	10.6
	Jun	Tillering-Heading	0.0	5.0	20.0
	Jul	Heading-Grain filling	-2.2	5.0	20.0
	Aug	Grain filling - Maturity	-2.2	5.0	23.5

Supplementary Table 5. The meteorological site ID number used in Northern Regional Performance Nursery (NRPN)

**Nursery**	**Country**	**State**	**Site**	**ID number**
NRPN	Canada[1]	Alberta	Lethbridge	Lethbridge
	USA[2]	Idaho	Aberdeen	USC00100010
	USA	Idaho	Tetonia	USC00109065
	USA	Iowa	Ames	USC00130200
	USA	Minnesota	Rosemount	USC00217107
	USA	Minnesota	Waseca	USC00218692
	USA	Montana	Bozeman	USC00241044
	USA	Montana	Moccasin	USC00245761
	USA	Nebraska	Alliance	USC00250130
	USA	Nebraska	Lincoln	USC00254790
	USA	Nebraska	Mead	USC00255362
	USA	Nebraska	North Platte	USC00256075
	USA	Nebraska	Sidney	USC00257830
	USA	New Mexico	Clovis	USC00291939
	USA	North Dakota	Carrington	USC00321360
	USA	North Dakota	Casselton	USC00321408
	USA	North Dakota	Hettinger	USC00324178
	USA	North Dakota	Williston	USC00329430
	USA	South Dakota	Brookings	USC00391076
	USA	South Dakota	Highmore	USC00393838
	USA	South Dakota	Presho	USC00396790
	USA	South Dakota	Winner	USC00399367
	USA	Wyoming	Archer	USC00480270
	USA	Wyoming	Sheridan	USC00488160

[1] Data from MSC[15]

[2] Data from GHCND[16]

Supplementary Table 6. The meteorological site ID number used in Southern Regional Performance Nursery (SRPN)

Nursery	Country	State	Site	ID number
SRPN	USA[1]	Colorado	Akron	USC00050109
	USA	Colorado	Burlington	USC00051121
	USA	Colorado	Ft. Collins	USC00053005
	USA	Colorado	Julesburg	USC00054413
	USA	Colorado	Springfield	USC00057866
	USA	Colorado	Walsh	USC00058793
	USA	Colorado	Yellow Jacket	USC00059275
	USA	Idaho	Aberdeen	USC00100010
	USA	Illinois	Urbana	USC00118740
	USA	Iowa	Ames	USC00130200
	USA	Kansas	Cloby	USC00141699
	USA	Kansas	Garden City	USC00142980
	USA	Kansas	HAYS	USC00143527
	USA	Kansas	Hutchinson	USC00143930
	USA	Kansas	Manhattan	USC00144972
	USA	Kansas	Winfield	USC00148964
	USA	Missouri	Columbia	USW00003945
	USA	Montana	Bozeman	USC00241044
	USA	Nebraska	Alliance	USC00250130
	USA	Nebraska	Clay Center	USC00251680
	USA	Nebraska	Hemingford	USC00253755
	USA	Nebraska	Lincoln	USC00254790
	USA	Nebraska	Mead	USC00255362
	USA	Nebraska	North Platte	USC00256075
	USA	Nebraska	Sidney	USC00257830
	USA	New Mexico	Clovis	USC00291963
	USA	New Mexico	Farmington	USC00293142
	USA	Oklahoma	Altus	USC00340184
	USA	Oklahoma	Cherokee	USC00341724
	USA	Oklahoma	Goodwell	USC00343628
	USA	Oklahoma	Lahoma	USC00344950
	USA	Oklahoma	Stillwater	USC00348501
	USA	Oklahoma	Woodward	USC00349760
	USA	South Dakota	Brookings	USC00391076

USA	South Dakota	Highmore	USC00393838
USA	South Dakota	Presho	USC00396790
USA	South Dakota	Winner	USC00399367
USA	Texas	Bushland	USC00411267

[1] Data from GHCND[16]

Supplementary Table 7. The meteorological site ID number used in Hard Red Spring Wheat Uniform Regional Nursery (HRSWURN)

Nursery	Country	State	Site	ID number
HRSWURN	Canada[1]	Manitoba	Winnipeg	Winnipeg
	Canada	Saskatchewan	Saskatoon	Saskatoon
	Canada	Saskatchewan	Swift Current	Swift Current
	USA[2]	Idaho	Aberdeen	USC00100010
	USA	Idaho	Tetonia	USC00109065
	USA	Minnesota	Crookston	USC00211891
	USA	Minnesota	Morris	USC00215638
	USA	Minnesota	Saint Paul	USC00218450
	USA	Montana	Bozeman	USC00241044
	USA	Montana	Havre	USW00094012
	USA	Montana	Sidney	USC00247560
	USA	Nebraska	Mead	USC00255362
	USA	North Dakota	Carrington	USC00321360
	USA	North Dakota	Casselton	USC00321408
	USA	North Dakota	Fargo	USW00014914
	USA	North Dakota	Hettinger	USC00324178
	USA	North Dakota	Langdon	USC00324958
	USA	North Dakota	Minot	USC00325993
	USA	North Dakota	Williston	USC00329420
	USA	South Dakota	Brookings	USC00391076
	USA	South Dakota	Highmore	USC00393832
	USA	South Dakota	Redfield	USC00397052
	USA	South Dakota	Selby	USC00397545
	USA	South Dakota	Watertown	USW00014946
	USA	Washington	Lind	USC00454679
	USA	Washington	Pullman	USC00456789
	USA	Wisconsin	Madison	USW00014837
	USA	Wyoming	Powell	USC00487388
	USA	Wyoming	Sheridan	USC00488160
	USA	Wyoming	Torrington	USC00488995

[1] Data from MSC[15]

[2] Data from GHCND[16]

Supplementary Table 8. Climate models used in the study

Climate models	Model sources	Release year
ACCESS-ESM1-5	CSIRO	2019
BCC-CSM2-MR	BCC	2017
CNRM-CM6-1	CNRM-CERFACS	2017
CNRM-ESM2-1	CNRM-CERFACS	2017
GFDL-ESM4	NOAA-GFDL	2018
IPSL-CM6A-LR	IPSL	2017

Supplementary References

1. National agricultural Statistics Service. Available at https://quickstats.nass.usda.gov (verified 29 April 2022).
2. Statistics Canada. Available at https://www.statcan.gc.ca/eng/start (verified 29 April 2022).
3. Sacks, W. , Deryng, D., Foley, J., & Ramankutty, N. Crop planting dates: an analysis of global patterns. *Glob. Ecol. Biogeogr.* **19**, 607–620 (2010).
4. James, G., Witten, D., Hastie, T. & Tibshirani R. An Introduction to Statistical Learning: With Applications in R. 1st ed. 2013, Corr. 7th printing 2017 edition. Springer; (2013).
5. Tack, J., Barkley, A. & Nalley, L. Effect of warming temperatures on US wheat yields. *Proc. Natl. Acad. Sci. U.S.A.* **112**, 6931–6936. (2015).
6. Lobell, D., Schlenker, W., & Costa-Roberts, J. Climate trends and global crop production since 1980. *Science*. **333**, 616 (2011).
7. Lobell, D., & Ivan Ortiz-Monasterio, J. Impacts of day versus night temperatures on spring wheat yields: A comparison of empirical CERES model predictions in Three locations. *Agron. J.* **99**, 469–477(2007).
8. Asseng, S. et al. Uncertainty in simulating wheat yields under climate change. *Nat. Clim. Change* **9**, 827–832 (2013).
9. Wilcox, J. & Makowski, D. A meta-analysis of the predicted effects of climate change on wheat yields using simulation studies. *Field Crop Res.* **156**, 180–190 (2013).
10. Asseng, S. et al. Rising temperatures reduce global wheat production. *Nat. Clim. Change*, **5**, 143–147. (2015).
11. Narciso, G., Ragni, P. & Venturi, A., Agrometeorological Aspects of crops in Italy, Spain and Greece. Joint Research Centre, Commission of the European Communities, Brussels–Luxembourg (1992).
12. Shroyer, J., Mikesell, M. & Paulsen, G., Spring freeze injury to Kansas wheat. Agricultural Experiment Station and Cooperative Extension Service (1995).
13. Porter, J. & Gawith, M., Temperatures and the growth and development of wheat: a review. *Euro. J. of Agron.* **10**, 23–36 (1999).
14. Saiyed, M., Bullock, R., Sapirstein, D., Finlay, J. & Jarvis, K. Thermal time models for estimating wheat phenological development and weather-based relationships to wheat quality. *Can. J. Plant Sci.* **89**, 429–439 (2009).
15. MSC. Meteorological Service of Canada. Available at https://climate.weather.gc.ca/ (verified 17 July 2021).
16. Menne, M., Durre, I., Vose, R., Gleason, B. & Houston, T. An overview of the Global Historical Climatology Network-Daily Database. *J. Atmos. Ocean*

Technol., **29**, 897–910 (2012).
