# Supplementary material for: Climate change may outpace current wheat breeding yield improvements in North America
Source: Nat Commun. 2022 Sep 30;13:5591. doi: 10.1038/s41467-022-33265-1 (PMC9525655; doi:10.1038/s41467-022-33265-1)
Supplement: Supplementary file 2 — Reporting Summary [file 41467_2022_33265_MOESM2_ESM.pdf]

nature portfolio

Corresponding author(s): Tianyi Zhang

Last updated by author(s): Sep 1, 2022

Reporting Summary

Nature Portfolio wishes to improve the reproducibility of the work that we publish. This form provides structure for consistency and transparency in reporting. For further information on Nature Portfolio policies, see our Editorial Policies and the Editorial Policy Checklist.

Statistics

For all statistical analyses, confirm that the following items are present in the figure legend, table legend, main text, or Methods section.

n/a	Confirmed	
☐	☒	The exact sample size (n) for each experimental group/condition, given as a discrete number and unit of measurement
☐	☒	A statement on whether measurements were taken from distinct samples or whether the same sample was measured repeatedly
☐	☒	The statistical test(s) used AND whether they are one- or two-sided<br>*Only common tests should be described solely by name; describe more complex techniques in the Methods section.*
☒	☐	A description of all covariates tested
☒	☐	A description of any assumptions or corrections, such as tests of normality and adjustment for multiple comparisons
☐	☒	A full description of the statistical parameters including central tendency (e.g. means) or other basic estimates (e.g. regression coefficient) AND variation (e.g. standard deviation) or associated estimates of uncertainty (e.g. confidence intervals)
☐	☒	For null hypothesis testing, the test statistic (e.g. F, t, r) with confidence intervals, effect sizes, degrees of freedom and P value noted<br>*Give P values as exact values whenever suitable.*
☒	☐	For Bayesian analysis, information on the choice of priors and Markov chain Monte Carlo settings
☒	☐	For hierarchical and complex designs, identification of the appropriate level for tests and full reporting of outcomes
☒	☐	Estimates of effect sizes (e.g. Cohen's d, Pearson's r), indicating how they were calculated

Our web collection on statistics for biologists contains articles on many of the points above.

Software and code

Policy information about availability of computer code

Data collection	The raw data are available on Zenodo repository: https://doi.org/10.5281/zenodo.7047191. Source data for each figure are provided with this paper.
Data analysis	The codes are available on the Zenodo repository: https://zenodo.org/record/7018108.

For manuscripts utilizing custom algorithms or software that are central to the research but not yet described in published literature, software must be made available to editors and reviewers. We strongly encourage code deposition in a community repository (e.g. GitHub). See the Nature Portfolio guidelines for submitting code & software for further information.

Data

Policy information about availability of data

All manuscripts must include a data availability statement. This statement should provide the following information, where applicable:

- Accession codes, unique identifiers, or web links for publicly available datasets
- A description of any restrictions on data availability
- For clinical datasets or third party data, please ensure that the statement adheres to our policy

The raw data are available on Zenodo repository: https://doi.org/10.5281/zenodo.7047191. Source data for each figure are provided with this paper.

Field-specific reporting

Please select the one below that is the best fit for your research. If you are not sure, read the appropriate sections before making your selection.

☐ Life sciences ☐ Behavioural & social sciences ☒ Ecological, evolutionary & environmental sciences

For a reference copy of the document with all sections, see nature.com/documents/nr-reporting-summary-flat.pdf

Ecological, evolutionary & environmental sciences study design

All studies must disclose on these points even when the disclosure is negative.

Study description	Variety adaptation to future climate for wheat is important but lacks comprehensive understanding. Here, we evaluate genetic advancement under current and future climate using a dataset of wheat breeding nurseries in North America during 1960-2018. Results show that yields declined by 3.6% per 1oC warming for advanced winter wheat breeding lines, compared with -5.5% for the check variety, indicating a superior climate-resilience. However, advanced spring wheat breeding lines showed a 7.5% yield reduction per 1oC warming, which is more sensitive than a 7.1% reduction for the check variety, indicating climate resilience is not improved and may even decline for spring wheat. Under future climate of SSP scenarios, yields of winter and spring wheat exhibit declining trends even with advanced breeding lines, suggesting future climate warming could outpace the yield gains from current breeding progress. Our study highlights that the adaptation progress following the current wheat breeding strategies is challenging.
Research sample	The two dataset covers the major wheat growing area in North America, as shown in Fig.1. In the two datasets, the yield of check varieties represent the yield performance without variety advances, and the yield of advanced breeding lines each year reflect the actual wheat breeding effort
Sampling strategy	We use all available data pair for yield and climate.
Data collection	Tianyi Zhang and Yong He collected the following data from respective sources: the winter wheat breeding data are publicly available online at https://www.ars.usda.gov/plains-area/lincoln-ne/wheat-sorghum-and-forage-research/docs/hard-winter-wheat-regional-nursery-program/research/. The spring wheat breeding data are publicly available online at https://wheat.pw.usda.gov/GG3/germplasm#wheat. Raw data are available on Zenodo repository: https://doi.org/10.5281/zenodo.7047191
Timing and spatial scale	We started data-collection from Jun. 2018, and ended on April 2020.
Data exclusions	We did not exclude any raw data.
Reproducibility	The regression coefficients reported in the study have been validated by bootstrapping method. Replication of results can be done through rerunning the regression analysis.
Randomization	We use model, so randomizatioin is not relevant.
Blinding	Our data do not involve investigation on any participant. So this does not apply to our study.

Did the study involve field work? ☐ Yes ☒ No

Reporting for specific materials, systems and methods

We require information from authors about some types of materials, experimental systems and methods used in many studies. Here, indicate whether each material, system or method listed is relevant to your study. If you are not sure if a list item applies to your research, read the appropriate section before selecting a response.

Materials & experimental systems

n/a	Involved in the study
☒	☐ Antibodies
☒	☐ Eukaryotic cell lines
☒	☐ Palaeontology and archaeology
☒	☐ Animals and other organisms
☒	☐ Human research participants
☒	☐ Clinical data
☒	☐ Dual use research of concern

Methods

n/a	Involved in the study
☒	☐ ChIP-seq
☒	☐ Flow cytometry
☒	☐ MRI-based neuroimaging
